# Characterization and Antioxidant and Angiotensin I-Converting Enzyme (ACE)-Inhibitory Activities of Gelatin Hydrolysates Prepared from Extrusion-Pretreated Milkfish (*Chanos chanos*) Scale

**DOI:** 10.3390/md16100346

**Published:** 2018-09-22

**Authors:** Chun-Yung Huang, Yung-Hsiang Tsai, Yong-Han Hong, Shu-Ling Hsieh, Ren-Han Huang

**Affiliations:** 1 Department of Seafood Science, National Kaohsiung University of Science and Technology, No. 142, Haijhuan Rd., Nanzih District, Kaohsiung City 81157, Taiwan; yht@nkust.edu.tw (Y.-H.T.); slhsieh@nkust.edu.tw (S.-L.H.); 2 Department of Nutrition, I-Shou University (Yanchao Campus), No. 8, Yida Rd., Jiaosu Village, Yanchao District, Kaohsiung City 82445, Taiwan; yonghan@isu.edu.tw; 3 Department of Nursing, Mackay Medical College, No.46, Sec. 3, Zhongzheng Rd., Sanzhi District, New Taipei City 25245, Taiwan; lisa68850@gmail.com

**Keywords:** Alcalase, angiotensin-I-converting enzyme, antioxidant, *Chanos chanos*, enzyme digestion, extrusion, Flavourzyme, gelatin, gelatin hydrolysate

## Abstract

Fish gelatin hydrolysates have been shown to possess various biological activities due to their unique Gly-Pro-Y and Gly-X-Hyp sequences. In the current study, fish gelatin was extracted from non-extruded milkfish scale (FSG1) or extrusion-pretreated milkfish scale (FSG2); extracted gelatins were hydrolyzed with different combinations of Flavourzyme and Alcalase to give four different hydrolysates, namely: FSGH1 (FSG1 hydrolyzed with Flavourzyme), FSGH2 (FSG1 hydrolyzed with Alcalase + Flavourzyme), FSGH3 (FSG2 hydrolyzed with Flavourzyme), and FSGH4 (FSG2 hydrolyzed with Alcalase + Flavourzyme). The extrusion-pretreatment process enhanced the extraction yield of gelatin from fish scale. Sodium dodecyl sulfate-polyacrylamide gel electrophoresis (SDS-PAGE) and Fourier transform infrared (FTIR) analyses showed the extracts FSG1 and FSG2 possessed characteristics of gelatin. Moreover, the physicochemical characteristics of FSGH1–FSGH4 were examined by analyses of their degree of hydrolysis, amino acid composition, UV spectrum, FTIR spectrum, molecular weight, and RP-HPLC profile. Additional biological functional analyses showed that all of the studied gelatin hydrolysates FSGH1–FSGH4 possessed antioxidant activity dose-dependently as revealed by DPPH scavenging, ABTS scavenging, and reducing power analyses. In addition, FSGH2 and FSGH4 showed higher angiotensin-I-converting enzyme (ACE)-inhibitory activity as compared to FSGH1 and FSGH3. Taken together, FSGH2 and FSGH4 showed high antioxidant activity and potent anti-ACE activity. Due to the potential antioxidant and antihypertensive properties of FSGH2 and FSGH4, further research is needed to explore their possible use as natural supplementary raw materials in food and nutraceutical products.

## 1. Introduction

Reactive oxygen species (ROS) in the forms of superoxide anion (∙O_2_^−^), hydroxyl radical (∙OH), and hydrogen peroxide (H_2_O_2_) are metabolic products which may also be present in the environment. ROS interact with one another in biological systems. The uncontrolled generation of ROS often correlates directly with molecular markers of many disease conditions including atherosclerosis, diabetes, inflammation, neurodegenerative diseases, and cancer [1,2,3]. Consumption of dietary antioxidants from natural sources is potentially effective for promoting human health, as antioxidation is known to mitigate the adverse effects of ROS. However, there have also been numerous reports suggesting that dietary antioxidant nutritive supplements may exhibit prooxidant and cytotoxic properties under certain conditions, which depend on such factors as metal-reducing potential, chelating behavior, pH, solubility characteristics, dose, duration of administration, and other dietary components [4,5].

Hypertension is a worldwide problem of epidemic proportions that affects 15–20% of all adults [6]. Hypertension is identified as a cardiovascular risk factor, and is often called a ‘‘silent killer’’ because hypertensive individuals are often asymptomatic for years [7]. Angiotensin-I-Converting Enzyme (ACE) plays an important physiological role in regulating blood pressure. ACE catalyzes the conversion of angiotensin from an inactive decapeptide (Angiotensin-I) to a potent vasoconstrictor octapeptide (Angiotensin-II) and also inactivates antihypertensive vasodilator bradykinin [8]. Therefore, inhibition of ACE activity is considered to be a useful therapeutic approach for the treatment of hypertension. More than a dozen ACE inhibitors have been used extensively in the treatment of essential hypertension and heart failure in humans; these include alacepril, benazepril, captopril, cilazapril, enalapril, fosinopril, lisinopril, moexipril, perindopril, quinapril, ramipril, tandolapril, and zofenopril [8,9]. These ACE inhibitors are likely to induce various adverse reactions, which are related to their particular chemical structure or kinetics [10]. Therefore, there is growing interest in the development of inhibitors derived from natural compounds, which may be safer and perhaps also cheaper.

Large quantities of fish are harvested annually worldwide, and approximately 50% of protein-rich fish processing byproducts are discarded or used as fishmeal and animal feed without any attempt to recover the essential nutrients [11]. The fish waste is mainly composed of heads, bones, skin, fin, scale, viscera, and sometimes whole fish. Among these types of fish waste, fish skin, bone, fin, and scale were examined to assess the availability of collagen and gelatin, which could potentially be isolated [12,13,14]. Fish scale, as compared to fish skin, fin, and bone, has lower amounts of non-collagenous proteins, and thus it is a better option for extraction of collagen and gelatin. It has been shown that fish protein hydrolysates have a wide range of bioactive effects, which include antioxidant, antihypertensive, immunomodulatory, mineral binding, and antimicrobial activities [15]. Fish gelatin hydrolysate is particularly promising, as it has exhibited higher biological activities compared to hydrolysate derived from fish muscle proteins due to its unique Gly-Pro-Y and Gly-X-Hyp sequences [16,17]. Thus, the bioactivities of gelatin peptides are thought to be related to their unique amino acid compositions [16,17]. Milkfish (*Chanos chanos*) is an important aquacultured fish in the Indo-Pacific region, particularly the Philippines, Indonesia, and Taiwan [18]. According to data from the Fisheries Agency, Council of Agriculture, Executive Yuan, Taiwan, in 2017, the production of milkfish in Taiwan was 52,234 metric tons/year, and the production of milkfish scales was estimated to be 1567–2612 metric tons/year. Large quantities of milkfish scales produced in fishery plants are discarded as waste. Therefore, the objective of this study was to extract gelatin from extrusion-pretreated milkfish fish scale. The extrusion process has been reported to facilitate the extraction of gelatin from fish scale matrix and eliminate the offensive fishy odor [19]. The extracted gelatins were digested with Flavourzyme or Alcalase + Flavourzyme to obtain gelatin hydrolysates and their compositions; characteristics, antioxidant, and ACE-inhibitory activities were evaluated. To the best of the authors’ admittedly limited knowledge, this is the first report to evaluate the antioxidant and ACE-inhibitory activities of gelatin hydrolysates prepared from extrusion-pretreated milkfish scale. In addition, it would be useful to explore the possible application of fish gelatin hydrolysates as functional food materials.

## 2. Results and Discussion

### 2.1. Preparation of Gelatins (FSG1 and FSG2) from Extrusion-Pretreated Milkfish Scale

The proximate compositions of raw milkfish scale on a wet weight basis are presented in Table 1. Raw milkfish scale consisted of 9.82% ± 0.42% moisture, 53.6% ± 0.7% crude protein, 0.25% ± 0.04% crude lipid, 33.0% ± 0.2% ash, and 3.34% ± 0.18% carbohydrate. The ash in fish scale is considered to be mostly hydroxyapatite [20]. In addition, milkfish scale was predominantly composed of protein (59.4% protein, dry basis), with a higher proportion of protein compared with spotted golden goatfish (45.2% protein, dry basis) [21] and tilapia (49.4% protein, dry basis) [19], indicating that milkfish scale is a good source for the extraction of gelatin. The extraction yields of gelatin for FSG1 and FSG2 were 4.03 ± 0.05 and 11.4 ± 0.2 g/100 g, dry basis, respectively (Table 2). These data clearly show that the extrusion-pretreatment process can increase the extraction yield of gelatin to around 3–fold (11.4/4.03 ≈ 3), indicating that the extrusion-pretreatment process enhanced the extraction of gelatin from milkfish scale samples. In addition, previous studies suggested that yields of gelatin extracted from lizardfish (*Saurida* spp.) scales ranged from 9.1–10.9% (dry basis), which are similar to that of FSG2. However, these investigations used a high temperature (70–90 °C) and a long duration (1–5 h) for the extraction of gelatin [22]. Processes which require a high temperature and a long duration may have limited industrial use. In the present study, after the extrusion-pretreatment process, only a lower temperature (50 °C) and shorter duration (1 h) condition (Table 2) is needed for the extraction of gelatin, which may be economically valuable for industrial applications.

### 2.2. Physicochemical Properties of Extracted Gelatins for FSG1 and FSG2

**Figure 1:**
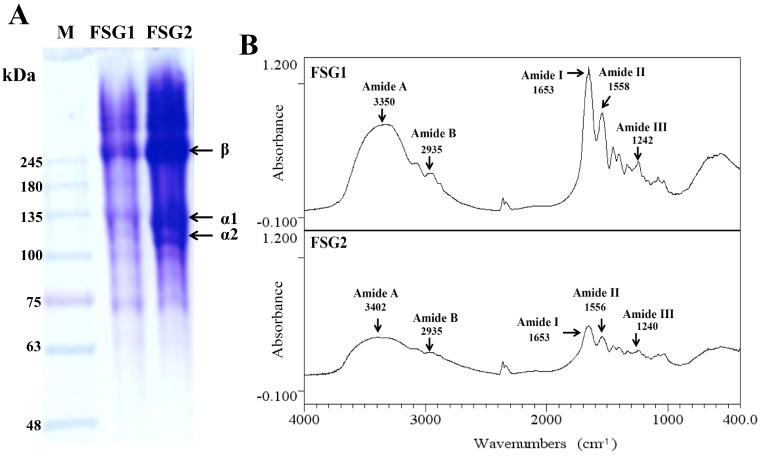
Characteristics of extracted gelatins for FSG1 and FSG2. (**A**) SDS-PAGE patterns of gelatins for FSG1 and FSG2. The first lane is protein marker (M). (**B**) FTIR spectra of gelatins for FSG1 and FSG2.

The physicochemical properties of the gelatin extracts FSG1 and FSG2 were characterized using sodium dodecyl sulfate-polyacrylamide gel electrophoresis (SDS-PAGE) and Fourier transform infrared (FTIR) analyses. The SDS-PAGE graphs of FSG1 and FSG2 presented in Figure 1A showed a similar electrophoretic pattern and both possessed two different α chains (α1 and α2) and their cross-linked β chains, which is characteristic of type-I collagen [23]. The results also suggest that the extrusion-pretreatment process did not seem to obviously alter the SDS-PAGE patterns of extracted gelatins. In addition, due to the higher gelatin extraction yield in FSG2, the band intensity of proteins from FSG2 was higher than that of FSG1 (Figure 1A). Previous studies showed that FTIR spectra of fish scale collagen possessed five major adsorption bands in the amide band region, including 1644–1653 cm^−1^ (amide I), 1541–1558 cm^−1^ (amide II), 1237–1239 cm^−1^ (amide III), 3304–3315 cm^−1^ (amide A), and 2922–2940 cm^−1^ (amide B) [13,24,25,26]. In general, the absorption peaks tended to shift due to the different extraction methods [21] or different collagen resources [13]. In the present study, FTIR spectra of FSG1 and FSG2 exhibited the characteristic peaks of amides I, II, and III, as well as amides A and B, as presented in Figure 1B. The major absorption bands for FSG1/FSG2 at 1653/1653 cm^−1^ (amide I), 1558/1556 cm^−1^ (amide II), 1242/1240 cm^−1^ (amide III), 3350/3402 cm^−1^ (amide A), and 2935/2935 cm^−1^ (amide B) were present, indicating that gelatins were extracted by this process. The extracted FSG1 and FSG2 were further utilized for the preparation of gelatin hydrolysates.

### 2.3. Preparation of Gelatin Hydrolysates (FSGH1, FSGH2, FSGH3, and FSGH4) from FSG1 and FSG2, and Characteristics of FSGH1, FSGH2, FSGH3, and FSGH4

**Figure 2:**
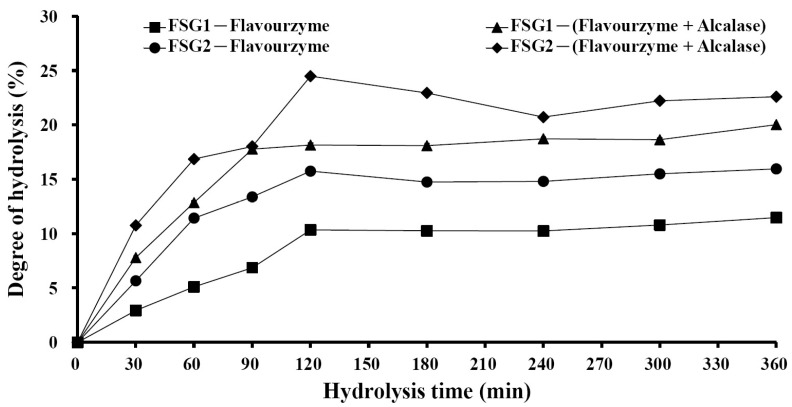
Degree of hydrolysis for various gelatin hydrolysates. The hydrolysis conditions were performed at pH 7.5, 50 °C, and for various times (0–360 min). FSG1-Flavourzyme: FSG1 digested with Flavourzyme; FSG1-(Alcalase + Flavourzyme): FSG1 digested with Alcalase + Flavourzyme; FSG2-Flavourzyme: FSG2 digested with Flavourzyme; FSG2-(Alcalase + Flavourzyme): FSG2 digested with Alcalase + Flavourzyme.

**Figure 3:**
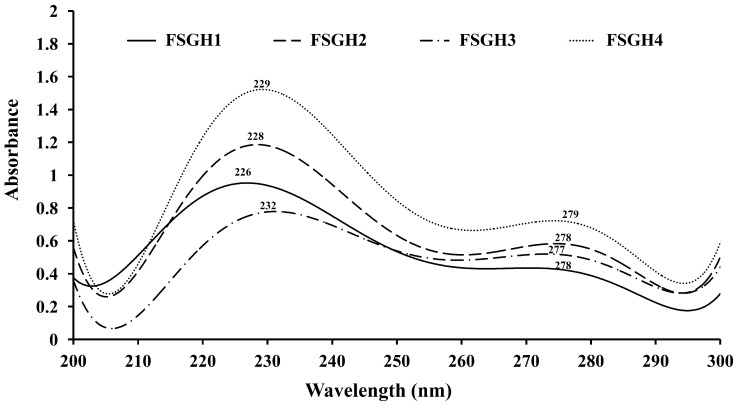
UV spectra of gelatin hydrolysates for FSGH1, FSGH2, FSGH3, and FSGH4.

**Figure 4:**
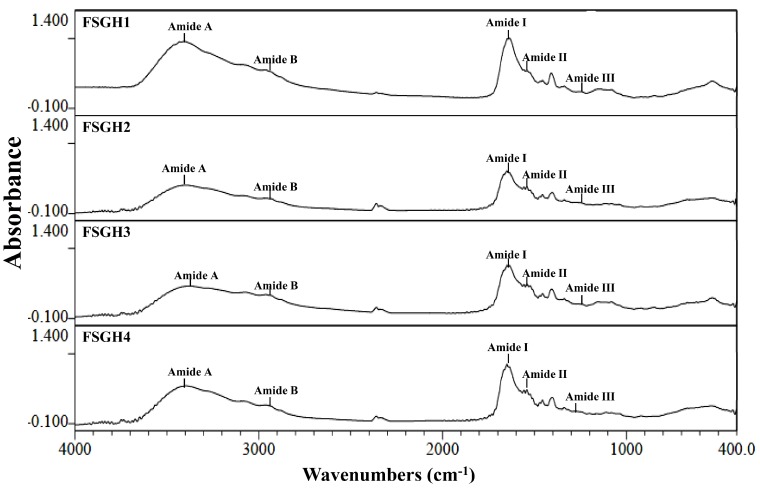
FTIR spectra of gelatin hydrolysates for FSGH1, FSGH2, FSGH3, and FSGH4.

**Figure 5:**
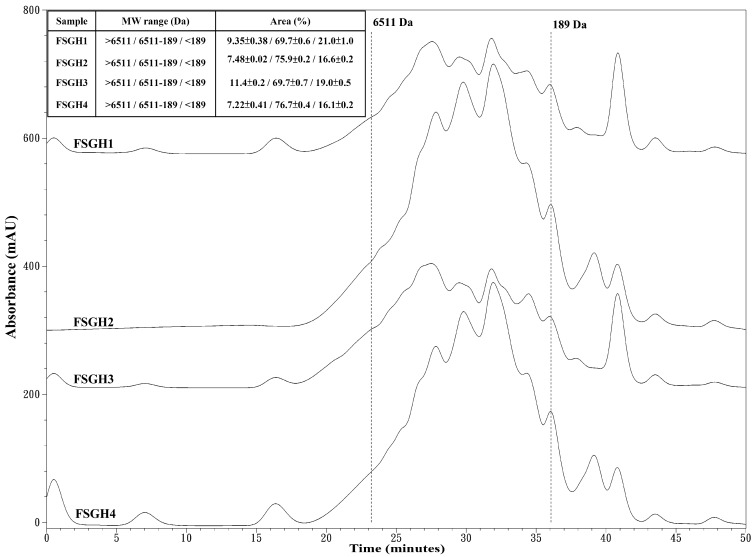
Size exclusion chromatographic profiles for FSGH1, FSGH2, FSGH3, and FSGH4. Aprotinin (6511 Da) and Gly-Gly-Gly (189 Da) were utilized as the standard proteins.

**Figure 6:**
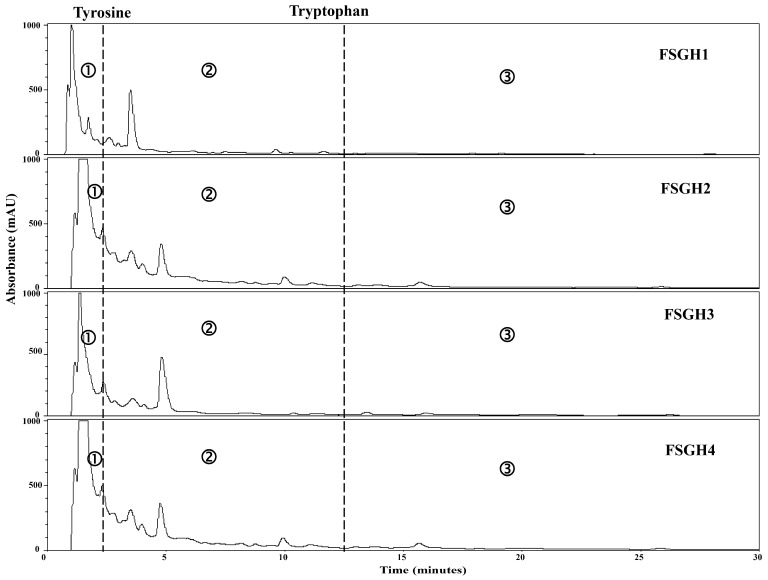
RP-HPLC profiles of four gelatin hydrolysates FSGH1, FSGH2, FSGH3, and FSGH4.

Enzymatic hydrolysis is widely used in the food industry, and this technique normally does not involve the use of any toxic chemicals or solvents. Thus, in the present study, the extracted gelatins FSG1 and FSG2 were hydrolyzed using industrial food-grade proteinases, namely Alcalase and Flavourzyme, to obtain gelatin hydrolysates. Alcalase is an endoprotease that has been used in a number of studies involving gelatin/collagen hydrolysis owing to its broad specificity as well as the high degree of hydrolysis that can be achieved in a relatively short time under moderate conditions [27]. Flavourzyme is an endo/exoprotease that has been used to produce a fish protein hydrolysate with acceptable functional properties [11]. Moreover, these two enzymes belong to a class of nonselective/specific or less specific proteases, and were therefore expected to be more effective at producing extensive hydrolysis of a protein molecule because they can cleave the larger peptide at many more positions than specific proteases, which may produce a protein hydrolysate with acceptable functional properties. Therefore, Alcalase and Flavourzyme were chosen in the current study. During the course of this investigation, we found that the total numbers of peptide bonds in FSG1 and FSG2 were 10.8 ± 0.1 and 11.2 ± 0.1 mequiv/g of gelatin, respectively, which were similar to a previously reported value (11.1 mequiv/g of gelatin) [28]. In addition, the degree of hydrolysis for various gelatin hydrolysates at different hydrolysis times is shown in Figure 2. It was found that the degree of hydrolysis reached a maximum at approximately 120 min for the different hydrolysis times tested. In addition, it was noted that Alcalase + Flavourzyme had a higher degree of hydrolysis than that of Flavourzyme alone. We also observed that FSG2 (extracted from extrusion-pretreated fish scale) was more susceptible to enzyme digestion as compared to FSG1 (extracted from non-extruded fish scale), indicating that extrusion pretreatment had a beneficial effect with respect to increasing hydrolysis efficiency in gelatin hydrolysates. Thus, the duration of hydrolysis utilized for subsequent hydrolysis experiments was 120 min. Previous research has suggested that the size, amount, amino acid composition, and amino acid sequences of gelatin and collagen-derived hydrolysates may influence their biological activities [27]. Thus, analyses of amino acid compositions, UV absorption, MW distribution, hydrophilic/hydrophobic peptide distributions, and amino acid sequences of protein hydrolysates were performed in the present study. The amino acid compositions of FSGH1, FSGH2, FSGH3, and FSGH4 were analyzed and the data are presented in Table 3. The results indicate that among all of the gelatin hydrolysates, glycine was the most abundant amino acid (approximately 288–326 residues/1000 residues). They were also found to contain large amounts of alanine, proline, glutamic acid/glutamine, aspartic acid/asparagine, arginine, and hydroxyproline, but small amounts of serine, histidine, threonine, tyrosine, cystine, valine, methionine, phenylalanine, isoleucine, leucine, lysine, tryptophan, and hydrolysine. In addition, unique amino acids in collagen were also found in these gelatin hydrolysates, i.e., hydroxyproline (approximately 26.7–49.9 residues/1000 residues) and hydroxylysine (approximately 12.5–22.1 residues/1000 residues). Examination of the amino acid compositions among these four gelatin hydrolysates revealed that enzymatic digestion of gelatin samples with Alcalase + Flavourzyme tended to increase the contents of hydrophobic amino acids such as phenylalanine, isoleucine, leucine, tryptophan, cystine, and valine as compared to those of gelatin samples digested with Flavourzyme alone. Several amino acids, such as tyrosine, methionine, histidine, lysine, and tryptophan, are well-known antioxidants [16,29]. A previous study suggested that the antioxidant activity of collagen and gelatin peptides is linked to the high content of hydrophobic amino acids, which could increase their solubility in lipids and therefore enhance their antioxidative activity [16]. In addition, ACE inhibitory protein hydrolysates and peptides have been shown to be related to a high concentration of hydrophobic amino acids, as well as to high proline levels [27,30,31]. Thus, it is necessary to further elucidate the biological functions of the gelatin hydrolysates, such as the antioxidant activity and ACE inhibitory activity of FSGH1, FSGH2, FSGH3, and FSGH4. Previous studies have shown that the protein might be collagen if there was a maximum absorption near 210–240 nm [32]. The UV absorption data of FSGH1, FSGH2, FSGH3, and FSGH4 are shown in Figure 3. All of the tested gelatin hydrolysates showed a maximum absorption peak at approximately 226–232 nm, which was related to the groups C=O, –COOH, and CONH_2_ in polypeptide chains of collagen [33]. The maximum absorption wavelength of protein near 280 nm can be attributed to the presence of aromatic amino acids such as tyrosine, tryptophan, and phenylalanine [34]. Here, a tiny level of absorbance was found for all gelatin hydrolysates tested at 280 nm, which was due to the low concentrations of aromatic amino acids in FSGH1, FSGH2, FSGH3, and FSGH4. The results were consistent with the findings in Table 3 which show that the contents of tyrosine, tryptophan, and phenylalanine in FSGH1, FSGH2, FSGH3, and FSGH4 were low. FTIR spectra of FSGH1–FSGH4 are presented in Figure 4. The major absorption bands (amides A, B, I, II, and III) of collagen could also be observed in the amide band region. It was also found that the FTIR spectra of FSGH1–FSGH4 (Figure 4) and those of FSG1–FSG2 (Figure 1B) were similar, indicating that although the enzyme digestion process could cleave the collagen molecule into smaller fragments, the characteristic functional groups in collagen could also be detected. The size of molecular weight (MW) is considered one of the most important determinants that affect the biological functions of protein hydrolysates. It has been reported that peptides consisting of 3–20 amino acids exhibit strong bioactivity [35]. As shown in Figure 5, the size exclusion chromatographic method provided the results of MW distribution in FSGH1–FSGH4. Of these four gelatin hydrolysates, the major MW distribution ranged from 189 to 6511 Da, accounting for approximately 69.7% of the total FSGH1 hydrolysate, 75.9% of the total FSGH2 hydrolysate, 69.7% of the total FSGH3 hydrolysate, and 76.7% of the total FSGH4 hydrolysate. These data also suggest that Alcalase + Flavourzyme together is more efficient at obtaining low-molecular-weight hydrolysates than Flavourzyme alone. Thus, the biological activities of gelatin hydrolysates, especially FSGH2 and FSGH4, warrant further characterization. RP-HPLC is an effective technique for separating peptides in protein hydrolysates and can determine the hydrophobic/hydrophilic peptide ratio [36]. The order in which hydrophilic and hydrophobic residues appear in a peptide has been shown to influence the elution profile of peptides [37]. The RP-HPLC elution profiles of four gelatin hydrolysates are presented in Figure 6. Two aromatic amino acids, tyrosine and tryptophan, were run separately and their retention times (2.34 min and 12.8 min, respectively) were used to divide the area under the chromatograms into three zones. Zone 1 consisted of peptides eluting before tyrosine (hydrophilic peptides). Zone 2 contained peptides eluting between tyrosine and tryptophan (low hydrophobic peptides), and zone 3 comprised peptides eluting after tryptophan (high hydrophobic peptides) [38]. Previous studies suggested that protein hydrolysate generated by protease, which gave a higher DH, presented more hydrophilic peptides [38]. Here, we also found that FSGH2 (digested by Alcalase + Flavourzyme) had higher DH (Figure 2) and higher hydrophilic peptides (Figure 6) than those of FSGH1 (digested by Flavourzyme). A similar pattern was observed for FSGH3 and FSGH4. Taken together, Alcalase + Flavourzyme exhibited a higher degree of hydrolysis than that seen using Flavourzyme alone. In addition, results from the LC-ESI/MS/MS analysis of FSGH1, FSGH2, FSGH3, and FSGH4 are shown in Table 4. The identification by LC-ESI/MS/MS of peptides of the most intense ions in the different hydrolysates showed homology with the collagen molecule, and glycine and proline were the two main amino acids present in the peptide sequences. In summary, FSGH1–FSGH4 showed characteristics of gelatin as examined by amino acid composition, UV spectrum, FTIR spectrum, and LC-ESI/MS/MS analyses. In addition, FSGH1–FSGH4 exhibited different MW and hydrophilic/hydrophobic peptide distributions. Thus, further investigation of their biological functions such as antioxidant and ACE inhibitory activities was conducted.

### 2.4. Antioxidant Activities of FSGH1, FSGH2, FSGH3, and FSGH4

**Figure 7:**
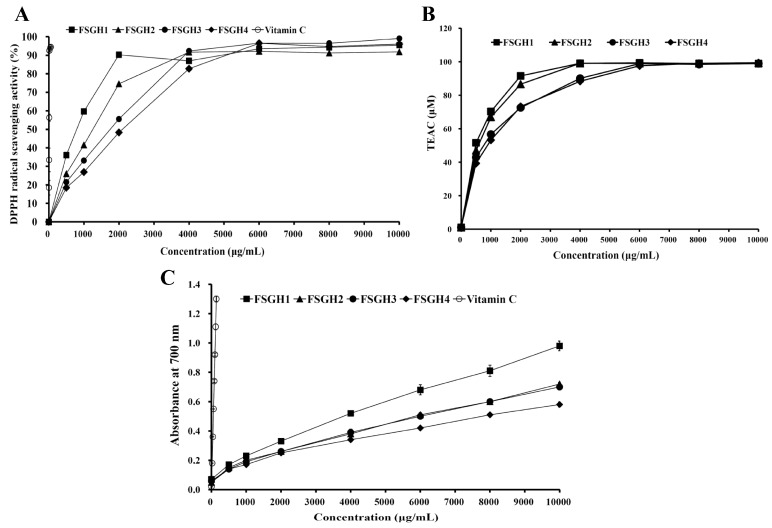
Antioxidant activities of gelatin hydrolysates. (**A**) DPPH radical scavenging activities for FSGH1, FSGH2, FSGH3, FSGH4, and vitamin C. (**B**) ABTS radical scavenging activities for FSGH1, FSGH2, FSGH3, and FSGH4. (**C**) Reducing power for FSGH1, FSGH2, FSGH3, FSGH4, and vitamin C.

In the present study, four gelatin hydrolysates FSGH1–FSGH4 were subjected to analyses with 2,2-diphenyl-1-picrylhydrazyl (DPPH), 2,2′-azino-bis (3-ethylbenzothiazoline-6-sulphonic acid) diammonium salt (ABTS), and reducing power analyses to determine their antioxidant activities. The scavenging effects of FSGH1–FSGH4 and vitamin C (as a positive control) on DPPH free radicals are shown in Figure 7A. All the gelatin hydrolysates FSGH1–FSGH4 exhibited DPPH scavenging properties in a dose-dependent manner. The IC_50_ values (concentration of sample capable of scavenging 50% of DPPH) of FSGH1, FSGH2, FSGH3, FSGH4, and vitamin C for DPPH radical scavenging activity were 800, 1280, 1790, 2100, and 10.9 μg/mL, respectively (Figure 7A). In general, the gelatin hydrolysates prepared from non-extrusion-pretreated fish scale (FSGH1 and FSGH2) had higher DPPH radical scavenging activity than that from extrusion-pretreated fish scale (FSGH3 and FSGH4). In addition, the gelatin hydrolysates obtained from combined enzyme digestion (Alcalase + Flavourzyme) (FSGH2 and FSGH4) had lower DPPH radical scavenging activity than that of gelatin hydrolysates obtained from digestion with Flavourzyme alone (FSGH1 and FSGH3). Thus, using Flavourzyme alone to obtain gelatin hydrolysate with high DPPH scavenging activity is feasible. A previous study revealed that the IC_50_ for the DPPH radical scavenging activity of gelatin hydrolysate obtained from thornback ray skin gelatin by treatment with *Bacillus subtilis* A26 protease was about 1980 μg/mL [38]. Another study reported that the IC_50_ for the DPPH radical scavenging activity of gelatin hydrolysates obtained from Nile tilapia scale gelatin by treatment with alcalase, pronase E, pepsin, or trypsin ranged from 660 to 1020 μg/mL [39]. These reported IC_50_ data were similar to our findings. ABTS radical assay is an excellent tool for determining the levels of hydrogen-donating antioxidants, and involves a process in which the radical is quenched to form an ABTS radical complex [40,41]. The ABTS^•+^ scavenging activities of the gelatin hydrolysates FSGH1–FSGH4 are shown in Figure 7B. All gelatin hydrolysates showed ABTS^•+^ scavenging activities in a dose-dependent pattern. We also found that the relative strengths of the ABTS^•+^ scavenging activities exhibited by the four gelatin hydrolysates at a concentration of 1000 μg/mL (*p* < 0.05) were FSGH1 (70.4 ± 1.6 μM) > FSGH2 (66.8 ± 1.2 μM) > FSGH3 (56.8 ± 0.8 μM) > FSGH4 (53.2 ± 0.6 μM). A similar pattern was also found for the DPPH scavenging properties of these four gelatin hydrolysates. The reducing power of a compound is generally related to the presence of reductones (antioxidants), which exert an antioxidant potential by donating a hydrogen atom, thereby breaking the free radical chain [42]. In the reaction system, antioxidant components in samples cause reduction of the Fe^3+^/ferricyanide complex to the Fe^2+^ form, and Fe^2+^ can be monitored by measuring the formation of Prussian blue at 700 nm. Figure 7C shows the reducing power of gelatin hydrolysates FSGH1–FSGH4 and vitamin C (as a positive control). All gelatin hydrolysates exhibited reducing power in a concentration-dependent manner. FSGH1 exhibited the highest reducing power, followed by FSGH2, FSGH3, and FSGH4 in descending order. Previous studies showed that the reducing power of thornback ray gelatin hydrolysates prepared by treatment with different proteolytic proteases of *B. subtilis* A26, *Raja clavata* crude alkaline protease extract, Alcalase, and Neutrase ranged from 0.25–0.85 at a concentration of 4000 μg/mL [38]. Our results showed that the reducing power of FSGH1–FSGH4 ranged from 0.30–0.55 at a concentration of 4000 μg/mL, which did not differ significantly from the result reported by Lassoued et al. [38].

### 2.5. ACE Inhibition Activity of FSGH1,FSGH2, FSGH3, and FSGH4 

The ACE inhibitory activities of FSGH1–FSGH4 were investigated, and the results are shown in Table 5. Among the captopril and four gelatin hydrolysates tested, captopril exhibited the highest inhibitory activity with an IC_50_ value of 0.002 ± 0.000 μg/mL, followed by FSGH2 with an IC_50_ value of 472 ± 12 μg/mL, FSGH4 with 547 ± 1 μg/mL, FSGH3 with 592 ± 0 μg/mL, and then FSGH1 with 762 ± 8 μg/mL. The observed results suggest that FSGH2 and FSGH4 possibly contained more potent ACE-inhibitory peptides than the other gelatin hydrolysates. Previous studies suggested that the difference in ACE inhibitory activity might be attributed to the differences in chain length and amino acid sequences of peptides as well as to their hydrophobicity [38]. Several studies also showed that hydrophobic amino acid residues (leucine, valine, alanine, tryptophan, tyrosine, proline, and phenylalanine) preferably bind with catalytic sites of ACE, hence acting as strong competitive ACE inhibitors [43]. In the present study, the high ACE-inhibitory activity in FSGH2 and FSGH4 might be attributed to their higher DH (Figure 2), lower MW (Figure 5), and higher hydrophobic amino acid contents (Table 3). Investigations have shown that although the peptides were potent inhibitors of ACE, they had limited application due to their lack of oral activity [44]. On the other hand, some studies have suggested that the antihypertensive effect of peptides (so-called prodrug peptides) might improve after oral administration due to the release of more active sequences by gastrointestinal digestion [45,46]. Therefore, the antihypertensive effect of orally administrated FSGH1–FSGH4 needs to be further characterized in future studies. Taken together, our data showed that all of the gelatin hydrolysates tested possessed effective anti-ACE activity. FSGH2 and FSGH4 exhibited relatively high anti-ACE activity and may therefore have the potential to exert an antihypertensive effect.

## 3. Materials and Methods

### 3.1. Materials and Chemicals

Fish scale from milkfish was obtained from a factory of a local fishery in Tainan, Taiwan. Fresh fish scale was kept on ice and transported to the laboratory immediately. Fish scale was mixed with 0.1 N NaOH to remove non-collagenous proteins [12], and the mixture was then washed with tap water until the pH reached 7.0. The resultant fish scale was dried at 50 °C until the moisture content was less than 10%. The dried fish scale was then milled into powder (<20 mesh) and stored in aluminum foil bags at room temperature until use. Flavourzyme, Alcalase, aprotinin, Gly-Gly-Gly, hydroxylysine, hydroxyproline, tyrosine, tryptophan, DPPH, potassium bromide (KBr), ABTS, 6-hydroxy-2,5,7,8-tetramethylchroman-2-carboxylic acid (Trolox), Prussian blue, o-pthaldialdehyde (OPA), bovine serum albumin (BSA), hippuryl-histidyl-leucine (HHL), hippuric acid, and captopril were purchased from Sigma-Aldrich (St. Louis, MO, USA). Sodium dodecyl sulfate (SDS), Coomassie Blue R-250, and N, N, N′, N′-tetramethylethylenediamine (TEMED) were procured from Bio-Rad Laboratories (Hercules, CA, USA). Superdex Peptide HR 10/30 column (300 mm × 10 mm ID) was purchased from GE Healthcare (Piscataway, NJ, USA). All other chemicals used were obtained from Sigma-Aldrich (St. Louis, MO, USA) and were all of analytical grade.

### 3.2. Extrusion-Cooking Procedure

The collagen (generally type I) in fish scale is tightly linked to hydroxyapatite [47], and both are difficult to separate [48]. To solve this problem, we employed a process that we previously developed involving extrusion-pretreatment primarily to damage the scale matrix so that it can be readily penetrated by water, thereby increasing the extraction yield of gelatin [19]. In brief, fish scale powder was used as the raw material and mixed with ddH_2_O until the final moisture content reached 27% as a preconditioning step. Extrusion cooking was performed using a model single-screw extruder (Tsung Hsing Food Machinery, Kaohsiung, Taiwan) equipped with a screw diameter of 74 mm, a screw length-to-diameter (L/D) ratio of 3.07:1, and a rounded die opening (5 mm) at the end of the extruder. Heating of the barrel was controlled by an electric heating element jacketing the barrel and thermal probe, and the barrel temperature was set at 135 °C. The screw speed was kept constant at 360 rpm. The feed rate was set at 11.4 kg/h to ensure stable operation of the extruder. The extrudate was collected at the die end and kept at 50 °C in a hot air oven for 30 min to remove extra moisture. After the extrusion-cooking procedure, the extrudate was ground into fine particles (<20 mesh), sealed in aluminum foil bags, and stored at 4 °C for further extraction experiments.

### 3.3. Determination of Protein Concentration

The Lowry assay was performed according to a method described previously [49]. Varying concentrations of stock BSA protein solution (1 mg/mL) were utilized for calibration.

### 3.4. Extraction of Gelatin from Non-extruded Fish Scale and Fish Scale Extrudate

The non-extruded fish scale powder or fish scale extrudate powder was soaked in ddH_2_O with a sample ratio of 1:10 (*w*/*v*) and shaken in a water bath at 50 °C for 1 h. The mixture was centrifuged at 10,200× *g* for 10 min, the supernatant was collected, determined its protein concentration by the Lowry method, lyophilized, and then two gelatins, namely FSG1 (non-extruded) and FSG2 (extrusion-pretreated), were obtained. The yield of gelatin was expressed as weight of protein extracted by hot water/weight of fish scale, dry basis, and calculated by Equation (1) below.

Yield (%) = [(protein content of supernatant (g/mL) × volume of supernatant (mL))/(weight of fish scale used (g), dry basis)] × 100(1)

**Figure 8:**
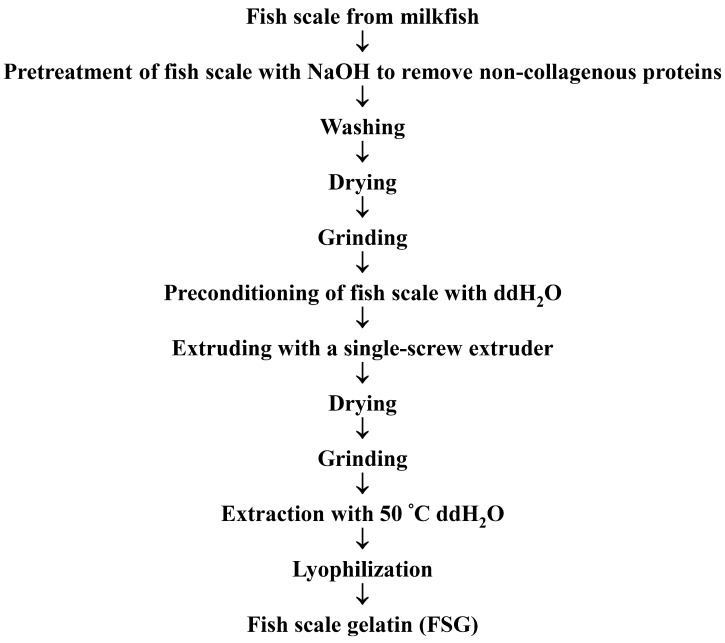
Flowchart of the extrusion pretreatment of milkfish scale and extraction of gelatin from milkfish scale extrudate.

A detailed description of the extrusion-pretreatment of fish scale and extraction of gelatin from fish scale extrudate is depicted in Figure 8. In addition, the extrusion variables and water extraction parameters are given in Table 2.

### 3.5. Chemical Composition Analyses

The determinations of the moisture, fat, ash, and crude protein were performed using the following AOAC (1984) [50] procedures: moisture (%) was measured by drying samples in an oven at 103 °C for 8 h; crude fat (%) was obtained gravimetrically after Soxhlet extraction with petroleum ether; crude ash (%) was determined by incineration in a muffle furnace at 580 °C for 8 h; crude protein was measured by the Kjeldahl method after acid digestion; and for the crude protein content calculation, a conversion factor of 5.95 was used [25].

### 3.6. SDS-PAGE

SDS-PAGE was performed according to the method described by Laemmli (1970) [51]. A 4% stacking gel and 7% separating gel were used. After electrophoresis, the gels were stained with Coomassie Blue R-250 dye in methanol:acetic acid:water solution (5:1:4, by volume) and destained in methanol:acetic acid:water solution without dye (1:1:8, by volume).

### 3.7. Fourier Transform Infrared (FTIR) Spectroscopy

Two milligrams of protein powder were ground evenly with approximately 100 mg KBr until particles measured < 2.5 μm in size. The transparent KBr pieces were made at 500 kg/cm^2^. The FTIR spectra were obtained using a FT-730 spectrometer (Horiba, Japan). The signals were automatically collected using 32 scans over the range of 4000 to 400 cm^−1^ at a resolution of 2 cm^−1^ and were compared to a background spectrum collected from the KBr alone at room temperature.

### 3.8. Preparation of Gelatin Hydrolysates

For digestion of fish scale gelatin, two proteases, namely, Alcalase and Flavourzyme, were used. Alcalase—an alkaline bacterial protease produced from *Bacillus licheniformis*—is a liquid preparation of serine endopeptidase that has a broad specificity and can hydrolyze most peptide bonds with higher specificity for aromatic (Phe, Trp, and Tyr), acidic (Glu), sulfur-containing (Met), aliphatic (Leu and Ala), hydroxyl (Ser), and basic (Lys) amino acid residues [52]. Flavourzyme—a protease preparation from Aspergillus oryzae—possesses very broad specificity and has eight enzymes, including two aminopeptidases, two dipeptidyl peptidases, three endopeptidases, and one α-amylase, that have been identified from Flavourzyme preparation [52]. Gelatin (2 g) was dissolved in a 100 mL 0.1 M sodium phosphate buffer solution, hydrolyzed with Flavourzyme (the ratio of protein to Flavourzyme was 100:6 (*w*/*w*)) or with Alcalase + Flavourzyme (the ratio of protein to Alcalase was 100:6 (*w*/*w*) and the ratio of protein to Flavourzyme was also 100:6 (*w*/*w*)) for 2 h at 50 °C in a batch reactor, heated at 98 °C for 10 min to inactivate the protease, then cooled by cold flowing water, and finally centrifuged at 1200× *g* for 15 min. The supernatant was lyophilized and the resulting hydrolysates were utilized for further experiments. Table 3 summarizes the operational variables for enzymatic digestion of FSG1 (digested with Flavourzyme or Alcalase + Flavourzyme) and FSG2 (digested with Flavourzyme or Alcalase + Flavourzyme). The gelatin hydrolysates were separated through an ultrafiltration membrane with a molecular weight cut-off of 3 kDa, and the 3 kDa permeate fractions were collected to obtain four gelatin hydrolysates, namely FSGH1, FSGH2, FSGH3, and FSGH4.

### 3.9. Evaluation of Degree of Hydrolysis

The DH of samples was determined by the OPA method [28] with slight modifications. The OPA assay was carried out by adding 3 mL of hydrolysate solution (or standard solution) to 3 mL of the OPA reagent. The absorbance of the mixed solution was measured at 340 nm by a UV spectrophotometer (UH5300 Spectrophotometer, Hitachi, Tokyo, Japan) after 5 min. The amount of free amino groups in the hydrolysate was calculated as serine-NH2 moieties using L-serine as the standard. The total number of primary amino groups in the gelatin sample was determined by acid hydrolysis of gelatin (6 N HCl at 110 °C for 24 h), assuming complete hydrolysis of all peptide bonds of the protein was achieved during acid digestion. The % DH was calculated according to following Equation (2).

%DH = ((NH_2_-Tx − NH_2_-To)/(NH_2_-Total − NH_2_-To)) × 100 (2)

NH_2_-To = Number of free -NH_2_ groups at 0 min of hydrolysis;

NH_2_-Tx = Number of free -NH2 groups in the supernatant after x minutes of protease-catalysed hydrolysis for each experimental point;

NH_2_-Total = Number of -NH2 groups resulting due to acid hydrolysis (complete hydrolysis of protein is assumed).

### 3.10. Determination of Amino acid Composition

A 0.01 g sample of protein was hydrolyzed in 0.6 mL of 6 M HCl in an evacuated and sealed tube at 110 °C for 24 h. The hydrolysate was dried at 65 °C under vacuum. Dry hydrolysate was dissolved in 2 mL of 0.25 M sodium citrate buffer (pH 2.2) and then filtered through 0.45-μm PVDF filters. A Shimadzu LC-10A high-performance liquid chromatography system (Shimadzu, Kyoto, Japan) equipped with a dual-pump LC-10AT binary system (Shimadzu, Kyoto, Japan), a fluorescence detector RF-10AXL (Shimadzu, Kyoto, Japan), and a Shim-pack AMINO-Na column (100 mm × 6.0 mm) was used to conduct the analysis. Amino acid analysis was done using pre-column fluorescence derivatization with OPA. The individual amino acid content was based on the area of the corresponding peak on the elution curves of the samples and standards, as determined by software (Sigma-Aldrich, St. Louis, MO, USA), and the sample’s amino acid composition was expressed on the basis of residues per 1000 total residues. The contents of methionine and cystine and/or cysteine were determined by HPLC analysis after converting them into methionine sulfone and cysteic acid, respectively (AOAC, 1984) [50]. Determination of tryptophan was also performed by HPLC analysis after alkaline hydrolysis (AOAC, 1984) [50].

### 3.11. Ultraviolet (UV) Absorption Spectrum

The UV absorption spectra of samples were studied following a method reported previously [53] with slight modifications. The extracted gelatin samples (1 mg) were dissolved in 1 mL of 0.5 M acetic acid. The gelatin solution was placed in a quartz cell with a path length of 10 mm. The clarified gelatin samples were subjected to absorbance at wavelengths between 200 and 300 nm at a scan speed of 10 nm per second with an interval of 0.1 nm. All spectra were obtained using a UV–visible spectrophotometer (UH5300 Spectrophotometer, Hitachi, Tokyo, Japan).

### 3.12. Molecular Weight Analysis

The molecular weight analysis of the gelatin hydrolysates was conducted using a Superdex Peptide HR 10/30 column (300 mm × 10 mm ID, GE Healthcare, Piscataway, NJ, USA) with a Shimadzu HPLC system (Shimadzu, Kyoto, Japan). The chromatography conditions were: eluent 0.02 M sodium phosphate and 0.25 M sodium chloride, at pH 7.20; flow rate 0.5 mL/min, sample concentration 1%; injection volume 45 μL; temperature 25 °C; and wavelength 214 nm. Standard proteins used for calibration of MW are aprotinin (6511 Da) and gly-gly-gly (189 Da).

### 3.13. Reversed-Phase High-Performance Liquid Chromatography (RP-HPLC)

The hydrophobicity of peptides from gelatin hydrolysates was studied using reversed-phase HPLC according to a previous method [38]. A Shimadzu HPLC system (Shimadzu, Kyoto, Japan) equipped with a Symmetry C18 (3 × 100 mm, 2.7 μm) from Nucleoshell (Macherey-Nagel, Düren, Germany) was used to conduct the analysis. Solvent A was TFA in double-distilled water (0.1%, *v*/*v*) and solvent B contained TFA (0.085%, *v*/*v*) in acetonitrile (ACN):double-distilled water (60:40, *v*/*v*). Both mobile phases A and B were filtered through a 0.45 μm nylon membrane filter and degassed prior to any analytical run. Peptides were first eluted with 100% solvent A for 2 min, followed by a linear gradient from 0 to 25% of solvent B for 40 min at a flow rate of 0.5 mL/min. The separation was monitored at a wavelength of 214 nm.

### 3.14. LC-ESI/MS/MS and Data Analyses

An UltiMate 3000 RSLCnano LC Systems (Thermo Fisher Scientific, Waltham, MA, USA) connected to a TripleTOF^®^ 6600 System (Applied Biosystems Sciex, Framingham, MA, USA) equipped with a nanoelectrospray ion source was used (Proteomic MS Core Laboratory, National Chung Hsing University, Taiwan). The hydrolysates (dissolved in ddH_2_O) were desalted on a C18 trap column (100 μm × 2 cm nanoViper, 3 μm, 100 Å, Thermo Fisher Scientific) for 4.5 min at a flow rate of 10 μL/min. The samples were then dried and redissolved in ddH_2_O containing 0.1% FA. Next, aliquots (10 μL) were injected using an autosampler. The sample was subsequently separated by a C18 resolving column (75 μm I.D. × 25 cm nanoViper, 2 μm, 100 Å, Thermo Fisher Scientific) at a flow rate of 300 nL/min. The mobile phases consisted of water with 0.1% formic acid (A) and 100% acetonitrile with 0.1% formic acid (B), respectively. Separation of the peptides was accomplished using a linear gradient of 5% to 30% B over 90 min, followed by 30% to 60% for 6 min, and 60% to 90% for another 6 min. The mass spectrometer was operated in the information-dependent acquisition (IDA) mode in which the initial MS scan recorded the mass to charge (*m*/*z*) ratios of ions over the mass range from 350 to 1500 Da, and then the 20 most abundant ions were automatically selected for subsequent collision-activated dissociation. All MS/MS data were searched against the UniProtKB/Swiss-Prot database using the Mascot program (Matrix Science, Version 2.3.0, London, United Kingdom). The following search parameters were used: “none” enzyme, oxidation (M), oxidation (HW), and deamidated (NQ); peptide mass tolerance ±0.05 Da; fragment mass tolerance ±0.03Da.

### 3.15. DPPH Radical Scavenging Activity

The DPPH radical scavenging activity was determined by a previously described method [54]. In brief, 50μL was added to 200 μL 0.1 mM DPPH solution (in methanol). The mixture was vortexed for 1 min and left in the dark for 30 min at room temperature. The absorbance of all sample solutions was measured at 517 nm using an ELISA reader (PowerWave 340, Bio-Tek Instruments, Winooski, VT, USA). The scavenging activity of DPPH radicals was calculated by Equation (3):

Scavenging activity (%) = (1 − A_sample_/A_control_) × 100 (3)

where A_control_ represents absorbance of the methanol solution of DPPH without the sample and A_sample_ is absorbance of the methanol solution of DPPH with tested samples.

### 3.16. ABTS Radical Scavenging Activity

The ABTS radical scavenging activity was determined according to a previous method [55]. The ABTS reagent was generated by mixing 5 mL of 7 mM ABTS solution with 88 μL of 140 mM potassium persulfate in the dark at room temperature for 16 h to allow the completion of radical generation. The solution was diluted with 95% ethanol so that its absorbance at 734 nm was adjusted to 0.70 ± 0.05. To determine the scavenging activity, 100 μL ABTS reagent was mixed with 100 μL of various sample solutions. The mixture was allowed to react at room temperature for 6 min and absorbance was measured at 734 nm using an ELISA reader (PowerWave 340, Bio-Tek Instruments, Winooski, VT, USA). The blank was prepared in the same manner, except that distilled water was used instead of the sample. The scavenging activity of ABTS radicals was calculated by Equation (4):

Scavenging activity (%) = (1 − A_sample_/A_control_) × 100 (4)

where A_control_ represents absorbance of ABTS without the sample and A_sample_ is absorbance of ABTS with tested samples. Trolox was prepared as a standard. A calibration curve of scavenging percentage against different concentrations of Trolox standard was prepared. The ABTS radical scavenging activity of samples was expressed as Trolox equivalent antioxidant capacity (TEAC), which represents the concentration (μM) of Trolox.

### 3.17. Reducing Power Assay

Reducing power was measured according to a previous method [56]. Briefly, 0.5 mL of the sample was mixed with 0.5 mL of phosphate buffer (0.2 M, pH 6.6) and 0.5 mL of potassium ferricyanide (1%). The mixture was incubated at 50 °C for 20 min, and 0.5 mL of trichloroacetic acid (10%) was added to the reaction followed by a centrifugation step (970× *g* for 10 min). Finally, 0.5 mL of the supernatant solution was mixed with 0.5 mL of ddH_2_O and 0.1 mL of FeCl_3_ (0.1%), and was then stood for 10 min. The absorbance was measured at 700 nm using an ELISA reader (PowerWave 340, Bio-Tek Instruments, Winooski, VT, USA).

### 3.18. ACE-Inhibiting Activity

The ACE-inhibiting activities of samples were assayed by measuring the concentration of hippuric acid liberated from HHL according to a previous method [29], with some modifications. For each assay, 50 μL of sample solution with 50 μL of ACE solution (a purified enzyme from rabbit lung) (60 mU in sodium borate buffer, pH 8.3) were pre-incubated at 37 °C for 20 min and then incubated with 200 μL of substrate (0.67 mM HHL in 0.05 M sodium borate buffer at pH 8.3) at 37 °C for 60 min. The reaction was then stopped by adding 250 μL of 1 N HCl. Hippuric acid concentration was determined using a Shimadzu HPLC system (Shimadzu, Kyoto, Japan) with an Inspire C18 column (4.6 mm × 250 mm, 5 μm) (Dikma Technologies Inc., Lake Forest, CA, USA). 

### 3.19. Statistical Analysis

Experiments were performed at least three times. All data are presented as mean ± standard deviation (SD). Statistical evaluation of data was performed by Student’s *t*-test or one-way analysis of variance (ANOVA), followed by Duncan’s Multiple Range tests. The differences were considered to be statistically significant if *p* < 0.05.

## 4. Conclusions

In this investigation, we found that an extrusion-pretreatment process increased the extraction yield of gelatin from milkfish scale samples. Moreover, FSG2 (extracted from extrusion-pretreated fish scale) was more susceptible to enzyme digestion as compared to FSG1 (extracted from non-extruded fish scale), indicating that extrusion pretreatment enhanced the hydrolysis efficiency of gelatin hydrolysates. Application of these findings could be economically beneficial in the food and fishery industries. Four gelatin hydrolysates FSGH1–FSGH4 showed unique characteristics as revealed by degree of hydrolysis, amino acid composition, UV spectrum, FTIR spectrum, MW, RP-HPLC profile, and LC-ESI/MS/MS analyses. Biological functional analyses revealed that all of the studied gelatin hydrolysates FSGH1–FSGH4 showed antioxidant and anti-ACE activities. The gelatin hydrolysates obtained from combined enzyme digestion (Alcalase + Flavourzyme) had lower antioxidant activities than those obtained from digestion with Flavourzyme alone, indicating that the use of Flavourzyme alone to obtain gelatin hydrolysate with high antioxidant activity is feasible. Among FSGH1–FSGH4, FSGH2 and FSGH4 showed high anti-ACE activity, which might be attributed to their higher DH, lower MW, and higher hydrophobic amino acid contents. As such, these gelatin hydrolysates may have potential as supplementary raw materials in the food and nutraceutical industries. In addition, our findings provide a method of reusing fish byproduct waste, which might be of value in the fishery industry. Further in vivo experiments on FSGH2 and FSGH4 regarding their antioxidant and antihypertensive capacities are warranted.

## Figures and Tables

**Table 1:** Proximate composition of milkfish (*Chanos chanos*) scale.

Proximate Analysis (%)	*C. chanos*
Moisture	9.82 ± 0.42 ^1^
Crude protein	53.6 ± 0.7
Crude lipid	0.25 ± 0.04
Ash	33.0 ± 0.2
Carbohydrate	3.34 ± 0.18

^1^ Values are mean ± SD (*n* = 3).

**Table 2:** Extrusion variables, extraction variables, and extraction yields of fish scale gelatins for FSG1 and FSG2.

**Variables of Extrusion**	**FSG1**	**FSG2**
Preconditioning solvent	—	ddH_2_O
Moisture content of fish scale powder (%)	—	27
Feed supply (kg/h)	—	11.4
Die diameter (mm)	—	5
Screw speed (rpm)	—	360
Barrel temperature (°C)	—	135
**Variables of Water Extraction**	**FSG1**	**FSG2**
Extraction temperature (°C)	50	50
Extraction time (h)	1	1
**Extraction Yield of Gelatins**	**FSG1** ^2^	**FSG2** ^2^
Extraction yield (%) ^1^	4.03 ± 0.05	11.4 ± 0.2 ***

—, not adopted. ^1^ The values are expressed as g protein/100 g fish scale, dry basis. ^2^ Values are mean ± SD (*n* = 3); ***, *p* < 0.001.

**Table 3:** Variables applied in preparing gelatin hydrolysates and amino acid compositions of FSGH1, FSGH2, FSGH3, and FSGH4.

**Process Variables**	**FSGH1**	**FSGH2**	**FSGH3**	**FSGH4**
Gelatin source	FSG1	FSG1	FSG2	FSG2
Enzyme used	Flavourzyme	Alcalase + Flavourzyme	Flavourzyme	Alcalase + Flavourzyme
Digestion conditions	pH 7.5, 50 °C, 2 h	pH 7.5, 50 °C, 2 h	pH 7.5, 50 °C, 2 h	pH 7.5, 50 °C, 2 h
Ultrafiltration condition	<3 kDa	<3 kDa	<3 kDa	<3 kDa
**Amino Acid Composition**	**FSGH1**	**FSGH2**	**FSGH3**	**FSGH4**
Aspartic acid/asparagine	58.8 ± 1.5 ^c^	50.9 ± 5.0 ^b^	37.1 ± 0.9 ^a^	51.8 ± 1.7 ^b^
Glutamic acid/glutamine	89.9 ± 2.0 ^d^	67.7 ± 2.6 ^c^	28.6 ± 0.7 ^a^	39.8 ± 1.3 ^b^
Serine	23.5 ± 1.6 ^ab^	28.3 ± 12.0 ^b^	10.0 ± 0.2 ^a^	13.9 ± 0.5 ^ab^
Histidine	18.2 ± 1.6 ^a^	18.3 ± 1.0 ^a^	27.9 ± 3.0 ^b^	19.1 ± 3.5 ^a^
Glycine	311 ± 1 ^ab^	292 ± 5 ^ab^	326 ± 21 ^b^	288 ± 21 ^a^
Threonine	22.5 ± 0.3 ^b^	27.7 ± 3.6 ^c^	9.25 ± 0.22 ^a^	12.9 ± 0.4 ^a^
Arginine	46.0 ± 5.0 ^a^	46.4 ± 2.6 ^a^	49.8 ± 3.3 ^a^	52.2 ± 3.6 ^a^
Alanine	134 ± 2 ^a^	140 ± 3 ^a^	151 ± 8 ^a^	143 ± 14 ^a^
Tyrosine	7.93 ± 0.24 ^a^	10.2 ± 1.9 ^a^	9.86 ± 0.25 ^a^	9.20 ± 0.26 ^a^
Cystine	2.04 ± 0.32 ^a^	6.71 ± 0.33 ^c^	3.83 ± 0.44 ^b^	6.16 ± 0.89 ^c^
Valine	23.2 ± 0.1 ^a^	37.5 ± 1.6 ^c^	30.5 ± 1.6 ^b^	39.4 ± 2.2 ^c^
Methionine	10.8 ± 1.2 ^a^	12.6 ± 0.6 ^a^	16.8 ± 2.5 ^b^	13.6 ± 2.0 ^ab^
Phenylalanine	16.5 ± 0.1 ^a^	32.1 ± 1.3 ^c^	19.9 ± 1.4 ^b^	32.3 ± 2.3 ^c^
Isoleucine	10.6 ± 0.2 ^a^	18.6 ± 1.3 ^b^	12.1 ± 0.9 ^a^	17.9 ± 1.5 ^b^
Leucine	21.6 ± 0.3 ^a^	39.2 ± 1.1 ^c^	29.0 ± 1.5 ^b^	42.3 ± 3.0 ^c^
Lysine	15.9 ± 0.2 ^ab^	13.5 ± 1.3 ^a^	18.5 ± 2.7 ^b^	15.8 ± 0.9 ^ab^
Tryptophan	6.26 ± 0.59 ^a^	9.74 ± 1.99 ^bc^	7.33 ± 0.79 ^ab^	10.7 ± 0.4 ^c^
Proline	126 ± 2 ^b^	102 ± 3 ^a^	146 ± 10 ^c^	125 ± 5 ^b^
Hydroxylysine	12.5 ± 0.4 ^a^	18.7 ± 1.0 ^c^	16.6 ± 0.5 ^b^	22.1 ± 0.5 ^d^
Hydroxyproline	42.0 ± 0.7 ^b^	26.7 ± 1.4 ^a^	49.9 ± 4.2 ^c^	44.9 ± 1.5 ^bc^
Total	1000	1000	1000	1000

^a–d^ Values are mean ± SD (*n* = 3); values with different letters within the same row differ significantly (*p* < 0.05).

**Table 4:** Identification by LC-ESI/MS/MS of a selection of the most intense ions corresponding to peptides in FSGH1, FSGH2, FSGH3, and FSGH4.

Hydrolysate	Protein Origin	Molecular Mass (+1)	Peptide Sequence	Modification
FSGH1	Collagen 1a1-like	1354.68	M.GPRGPPGPPGPSGPQ.G	
		1411.70	M.GPRGPPGPPGPSGPQG.F	
	Collagen alpha-5(VI) chain (Fragment)	1443.69	V.GPRGSPGPPGQPGPQG.F	1 Deamidated (NQ)
FSGH2	collagen alpha-1(I) chain-like	1072.55	M.GPRGPPGPPGPS.G	
		1354.68	M.GPRGPPGPPGPSGPQ.G	
		1401.73	P.AGPSGPRGPAGPAGPR.G	
		1411.70	M.GPRGPPGPPGPSGPQG.F	
	collagen alpha-1(I) chain	1370.71	A.GPRGLPGPPGSPGPQ.G	
	Collagen alpha-1(I) chain (Fragments)	1370.71	S.GPRGIPGPPGSPGPQ.G	
	collagen alpha-1(I) chain-like	1427.73	M.GPRGLPGPPGPSGPQG.F	
	Collagen alpha-1(IV) chain (Fragment)	1443.69	L.GPRGSPGPPGQPGPQG.P	1 Deamidated (NQ)
	Collagen alpha-4(IV) chain	1087.64	I.GPLGPLGPIGIP.G	
	Collagen alpha-1(III) chain (Fragment)	1329.64	P.GNPGSPGPPGPLGPQ.G	1 Deamidated (NQ)
	Collagen alpha-1(V) chain	1386.67	T.GPPGRSGPQGPPGPAG.E	1 Deamidated (NQ)
	Collagen	1174.54	S.DGAPGGPGAPGPAGP.Q	
	Collagen type IX alpha 1 chain	985.51	D.PGRGPPGPPGP.P	
	Putative fibril-forming collagen alpha chain-like protein (Fragment)	976.50	R.DGLPGPPGPIG.I	
	Putative Collagen iv alpha 1 chain (Fragment)	1245.58	L.NGAPGAPGGAPGPAGP.K	1 Deamidated (NQ)
	Collagen-like surface protein	1129.69	V.APVIPVAPVAPV.S	
	Collagen alpha-1(XXI) chain (Fragment)	974.56	E.GPPGIPGPIGL.P	
	Collagen alpha-1(XXVI) chain	1362.27	P.GPSGQNGRPGIPGAP.G	1 Deamidated (NQ)
	Collagen alpha-1, IV, chain precursor, putative	958.53	E.PGIPGIGPPGP.I	
	Collagen type IV alpha 6 chain	974.56	V.PGIGLPGPLGP.R	
	collagen alpha-5(IV) chain isoform X1	974.56	M.GPPGLPGPLGI.P	
	Collagen alpha-2(XI) chain	974.56	E.GPPGPIGPIGI.P	
	Collagen alpha-1(XXVIII) chain (Fragment)	974.56	F.GPPGLPGPIGL.P	
FSGH3	collagen alpha-1(I) chain-like	1354.68	M.GPRGPPGPPGPSGPQ.G	
		1411.70	M.GPRGPPGPPGPSGPQG.F	
	collagen alpha-1(VII) chain	994.50	A.GPAGAAGSPGPR.G	
	collagen alpha-1(XII) chain	1443.69	I.GSPGPRGQPGPPGPQG.E	1 Deamidated (NQ)
	Collagen alpha-1(V) chain	1386.67	T.GPPGRSGPQGPPGPAG.E	1 Deamidated (NQ)
	Collagen	1370.67	P.GPRGPPGPPGEAGQP.G	
		1427.70	P.GPRGPPGPPGEAGQPG.P	
	Collagen, type I, alpha 1a	1027.47	G.MPIPGPMGPM.G	
	Type 1 collagen alpha 1	1339.67	T.GFPGSAGRVGPPGPS.G	
	Collagen triple helix repeat-containing protein	1174.61	G.IQGVPGPQGPAGP.Q	
FSGH4	collagen alpha-1(I) chain-like	1044.50	R.GPPGPPGPSGPQ.G	
		1101.53	R.GPPGPPGPSGPQG.F	
		1354.68	M.GPRGPPGPPGPSGPQ.G	
		1411.70	M.GPRGPPGPPGPSGPQG.F	
	Collagen alpha-4(IV) chain	985.51	A.GPPGRPGPPGP.A	
	Collagen alpha-1(IV) chain (Fragment)	1258.61	L.GPRGSPGPPGQPGP.Q	1 Deamidated (NQ)
		1443.69	L.GPRGSPGPPGQPGPQG.P	1 Deamidated (NQ)
	Putative Collagen iv alpha 1 chain (Fragment)	1245.58	L.NGAPGAPGGAPGPAGP.K	1 Deamidated (NQ)
	Collagen, type XXVII, alpha 1 (Fragment)	1258.61	H.GPRGSPGPQGPPGP.P	1 Deamidated (NQ)
	Collagen type VII alpha 1 chain	976.50	P.LGDPGPPGPLG.P	
	Collagen type XVIII alpha 1 chain	1101.53	P.GPPGPPGPSGPAN.T	
	Collagen	963.44	G.GPGEPGPAGAPG.T	

**Table 5:** ACE inhibition activities for FSGH1, FSGH2, FSGH3, FSGH4, and captopril (as a positive control).

	IC_50_ Value ^1^ (μg/mL)
FSGH1	762 ± 8 ^e^
FSGH2	472 ± 12 ^b^
FSGH3	592 ± 0 ^d^
FSGH4	547 ± 1 ^c^
captopril	0.002 ± 0.000 ^a^

^1^ The IC_50_ value represents the concentration of each compound that inhibits ACE activity by 50%. ^a–e^ Values are mean ± SD (*n* = 3); values with different letters within the same column differ significantly (*p* < 0.05).
